# The role of cell-mediated immunity against influenza and its implications for vaccine evaluation

**DOI:** 10.3389/fimmu.2022.959379

**Published:** 2022-08-16

**Authors:** Yorick Janssens, Jasper Joye, Gwenn Waerlop, Frédéric Clement, Geert Leroux-Roels, Isabel Leroux-Roels

**Affiliations:** ^1^ Center for Vaccinology (CEVAC), Ghent University, Ghent, Belgium; ^2^ Center for Vaccinology (CEVAC), Ghent University Hospital, Ghent, Belgium

**Keywords:** influenza, cellular immunity, vaccine, clinical trial, correlate of protection, T-cell, CD4, CD8

## Abstract

Influenza vaccines remain the most effective tools to prevent flu and its complications. Trivalent or quadrivalent inactivated influenza vaccines primarily elicit antibodies towards haemagglutinin and neuraminidase. These vaccines fail to induce high protective efficacy, in particular in older adults and immunocompromised individuals and require annual updates to keep up with evolving influenza strains (antigenic drift). Vaccine efficacy declines when there is a mismatch between its content and circulating strains. Current correlates of protection are merely based on serological parameters determined by haemagglutination inhibition or single radial haemolysis assays. However, there is ample evidence showing that these serological correlates of protection can both over- or underestimate the protective efficacy of influenza vaccines. Next-generation universal influenza vaccines that induce cross-reactive cellular immune responses (CD4^+^ and/or CD8^+^ T-cell responses) against conserved epitopes may overcome some of the shortcomings of the current inactivated vaccines by eliciting broader protection that lasts for several influenza seasons and potentially enhances pandemic preparedness. Assessment of cellular immune responses in clinical trials that evaluate the immunogenicity of these new generation vaccines is thus of utmost importance. Moreover, studies are needed to examine whether these cross-reactive cellular immune responses can be considered as new or complementary correlates of protection in the evaluation of traditional and next-generation influenza vaccines. An overview of the assays that can be applied to measure cell-mediated immune responses to influenza with their strengths and weaknesses is provided here.

## 1 Introduction

**Figure 1:**
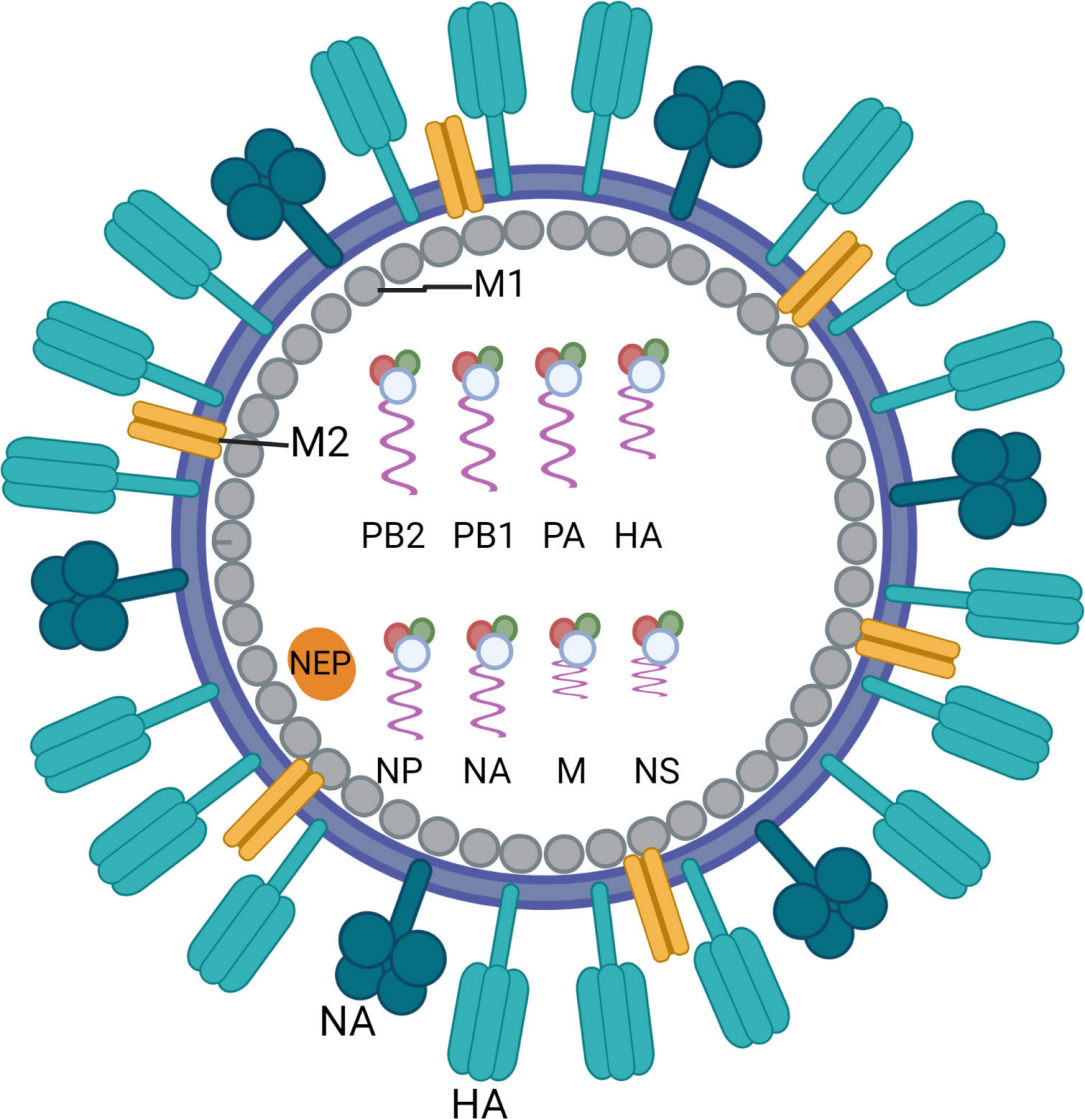
Schematic overview of the structure of an influenza virus particle. Both influenza A and influenza B viruses are enveloped negative-sense RNA viruses with genomes comprising eight single-stranded RNA segments located inside the virion. The three largest RNA segments encode the three subunits of the viral RNA-dependent RNA polymerases (PB1, PB2 and PA). The smaller RNA segments encode for haemagglutinin (HA), which mediates viral entry in epithelial cells by binding to sialic acid-containing receptors, and nucleoprotein (NP) which binds to the viral genome. The smaller RNA segments can encode for more than one protein. They mainly encode for neuraminidase (NA) which enables virus release from the host cell, matrix protein M1 (M1) and matrix protein M2 (M2), the non-structural protein (NS1) and the nuclear export protein (NEP). Figure created with BioRender.com.

Influenza viruses belong to the *Orthomyxoviridae*, a family of enveloped negative-sense single-stranded RNA viruses with a segmented genome. Three genera, influenza A, B and C, cause human respiratory disease of which influenza A and B are clinically most important. The influenza A viruses are further classified into subtypes according to the antigenicity of their major membrane glycoproteins, haemagglutinin (HA) and neuraminidase (NA). Since 1977 influenza A/H1N1 and A/H3N2 have been co-circulating and causing annual epidemics of varying severity (1). Influenza B viruses are not categorized into subtypes but are separated into two distinct genetic lineages (B/Yamagata and B/Victoria). Influenza B viruses from both lineages have co-circulated in most influenza seasons since the 1980s (2). The genomes of types A and B contain eight RNA segments encoding for surface proteins (HA, NA and matrix protein M2), RNA polymerase subunits (PA, PB1 and PB2), matrix protein M1, viral nucleoprotein (NP), a nonstructural protein (NS1) and a nuclear export protein (NEP) (**Figure 1**) (3). HA is the major target of host antibody responses elicited by natural infection or vaccinations that provide protection against influenza infection in humans. However, the lack of proofreading activity of the RNA-dependent RNA polymerase complex of influenza leads to point mutations in its genes. The accumulation of small changes over time within the antibody-binding sites of HA and NA results in viruses that are antigenically different and causes the emergence of variant viruses that can evade immune recognition. This phenomenon, called antigenic drift, is the main reason why people can catch the flu more than once. Antigenic drift is observed in both influenza A and B viruses, as opposed to another type of change that is called antigenic shift and is only seen in influenza A viruses. Antigenic shift is an abrupt, major change in the influenza A viruses that occurs through genetic reassortment of gene segments between different influenza viruses during co-infection of the same human or non-human (e.g. porcine) host cell. This may lead to the introduction of a new, potentially pandemic influenza A virus with a novel HA (and NA) against which humans have limited or no pre-existing immunity. The most recent “shift” occurred in the spring of 2009, when an H1N1 virus with a new combination of genes emerged to infect people and quickly spread, causing a pandemic.

Seasonal infections with influenza viruses affect people of all ages but most strongly children and older adults. Associated symptoms are generally mild and characterized by fever, sore throat, runny nose, cough, headache, muscle pain and fatigue but can also be more severe and in some cases lead to lethal pneumonia. Annual epidemics of influenza can result in approximately 1 billion infections leading to 3-5 million cases of severe illness and 300,000 to 500,000 deaths globally (1).

Annual influenza vaccination is the most effective way to prevent influenza infection and its complications. Many countries have adopted recommendations for annual influenza vaccination. Generally, these recommendations are primarily aimed at older adults and persons at risk of complications of influenza and less commonly at children, although vaccination of children can help reduce the incidence of severe virus infection in older adults as they are the main driver of influenza epidemics (1). Since the 2005/2006 season, the United States (US) has recommended influenza vaccination for all persons aged ≥6 months (4).

## 2 Current vaccines and correlates of protection

Until recently, annual vaccination was performed with trivalent inactivated influenza vaccines (TIV) containing 15 µg haemagglutinin (HA) of each of the three selected influenza strains (A/H1N1, A/H3N2 and one B lineage) which are administered intramuscularly. The influenza strains are selected for inclusion in a seasonal vaccine on the basis of predictions made from surveillance data acquired under the coordination of the WHO (Global Influenza Surveillance and Response System, GISRS). However, since there are two influenza B lineages circulating (B/Yamagata and B/Victoria), suboptimal vaccine protection can occur when the predominant circulating influenza B virus strain is from the alternate lineage to the B strain present in the vaccine (5, 6). The same is true when a mismatch occurs in influenza A strains (7–9). The recent introduction of inactivated quadrivalent influenza vaccines (QIV) containing both B strains was expected to alleviate the problem of mismatching between B lineages. A recent study however demonstrated that the use of QIV is not associated with an increased protection against any influenza B illness (10). In addition, due to COVID-19 measures taken as of March 2020 (e.g. social distancing, wearing masks, travel restrictions), the genetic diversity of influenza viruses has dramatically diminished with no observed isolations of the B/Yamagata lineage between April 2020 and August 2021. This could point to the global extinction of this lineage and may have favorable implications for future annual vaccine reformulations (e.g. inclusion of an extra A/H3N2 strain to minimize the risk of mismatch) (11). Inactivated vaccines can be manufactured by producing the virus strains in either embryonated eggs or eukaryotic cells or by applying recombinant DNA technologies in which solely the HA antigen is expressed in an insect cell-line using a baculovirus expression system (1). These vaccines mainly rely on the induction of HA-specific antibodies that prevent entry of the virions in the respiratory epithelial cells. To a lesser extent, NA specific antibodies are generated that interfere with the release of nascent viruses (12). In recent years there is growing evidence for antibody-dependent cellular cytotoxicity (ADCC) contributing to vaccine-induced protection (13). ADCC is a cell-mediated immune defense whereby specific antibodies that bind to membrane-surface antigens expressed on target cells also interact with Fc receptors on effector cells (e.g. Natural Killer (NK) cells) and thus lead to lysis of the target cell. Antibodies that elicit ADCC often target internal proteins of the virus such as M1 and NP (antigens that are not present in currently licensed vaccines) and are common in both healthy and infected adults. These antibodies could thus offer cross-reactive protection (14, 15). ADCC could be the main mechanism in which antibodies against conserved (but subdominant) epitopes work.

In 2003, the first live-attenuated influenza virus vaccine (LAIV) was licensed in the US as an intranasal spray. This vaccine is produced by re-assortment of the selected influenza virus strains with the cold-adapted A/Ann Arbor/6/1960 vaccine strain, which replicates efficiently at 25°C in the nasal passages but not at the higher temperatures deeper in the respiratory tract. This LAIV seems to have the advantage to induce mucosal IgA and more broadly protective immune responses in infants and children compared to inactivated vaccines (1, 16). However, pre-existing antibodies appear to have a negative impact on LAIV effectiveness in adults (12).

For many years, the evaluation of vaccine immunogenicity and licensure relied merely on the serological assessment of the immune response using the haemagglutination inhibition (HI) and the single radial haemolysis (SRH) assays. Every year, vaccine manufacturers had to do a small clinical trial involving around 100 participants including 50 subjects aged ≥60 years of whom pre- and post-vaccination serum samples had to be collected. The immunogenicity of the vaccine was then assessed based on three criteria that are shown in **Table 1** and were defined by the Committee for Medicinal Products for Human Use (CHMP). In order to be licensed, at least one of these criteria had to be met for a seasonal influenza vaccine while all criteria needed to be met for a pandemic vaccine (17).

**Table 1:** CHMP criteria for the evaluation of influenza vaccines.

	Adults (18-60 years)	Older adults (>60 years)
Seroprotection^1^	>70%	>60%
Seroconversion^2^	>40%	>30%
GMT increase^3^	>2.5	>2

The seroprotection cut-off is defined as ≥1:40 in the HI assay and >25 mm² in the SRH assay (1).

Seroconversion is defined as at least a 4-fold increase in titer after vaccination (2).

Ratio of pre- and post-vaccination geometric mean titers (3).

The cut-off values of >1:40 in the HI assay, >25 mm² in the SRH assay and a 4-fold increase of the HI antibody titers were considered as correlates of protection, meaning that these criteria are associated with a 50% risk reduction of infection or developing symptoms. However, the challenge study on which these criteria have been based dates back from 1972, was performed in healthy adults and did thus not consider populations at risk such as vulnerable children, older adults and immunocompromised patients (18). Many studies performed since then question the use of these serological criteria (12, 19). Indeed, a study performed in children demonstrated that a cut-off of 1:110 would be more appropriate to predict the 50% clinical protection rate (20). A meta-analysis by de Jong et al. showed a strong variation in HI titers required to obtain a 50% protection in adults (21). Studies in older adults are unfortunately scarce but one study demonstrated that 60% of infected older adults had titers ≥1/40 and 31% had titers ≥1/640. This shows that the HI titer of ≥1/40 cannot be applied as a correlate of protection in older adults (22). In addition, when a mismatch occurs between the vaccine and circulating strains, HI titers no longer correlate with protection (23). Therefore, other correlates of protection are needed to better predict protection rates. Recently, more and more studies point towards the use of cellular correlates of protection to complement serological parameters. Verschoor et al. analyzed IFN-γ and IL-10 secretion by virus-challenged peripheral blood mononuclear cells (PBMC) and observed that the fold increase of IFN-γ was significantly associated with protection (24). After oral vaccination with an adenovirus-based vaccine, the abundance of HA-specific plasmablasts and plasmablasts positive for integrin α4β7, phosphorylated STAT5, or lacking expression of CD62L at day 8 were significantly correlated with protection from developing viral shedding following H1N1 virus challenge (25). During the 2009 H1N1 pandemic, it was clear that persons with higher frequencies of pre-existing T-cells to conserved CD8 epitopes developed less severe illness (26). A human challenge study demonstrated that pre-existing CD4^+^ T-cells responding to internal influenza proteins were associated with lower virus shedding and less severe illness (27). Although vaccination is still considered the best tool to prevent influenza infection, vaccine effectiveness has become an issue of increasing confusion and debate. Several systematic reviews questioned the benefits of influenza vaccination for older adults (28–30). In addition, a growing body of evidence suggests that protection conveyed by a seasonal influenza vaccine may be reduced by vaccinations administered in prior seasons. Hoskins et al. were the first to report in 1979 that annual influenza vaccination conferred no long-term advantage and could even be disadvantageous (31). Over the past decade the Test Negative Design (TND), i.e. using a control group that tests negative for the pathogen, has been applied to evaluate vaccine effectiveness in many countries and a meta-analysis of TND studies revealed a low vaccine effectiveness for the A/H3N2 subtype (32). The Canadian Sentinel Practitioner’s Surveillance Network explored the effects of prior vaccination on current season’s vaccine effectiveness during epidemics in Canada between 2010-2011 and 2014-2015. The effects of repeat influenza vaccinations were consistent with the antigen distance hypothesis (i.e. negative interference from prior season’s vaccines on current vaccine) and may have contributed to the low vaccine effectiveness across recent A/H3N2 epidemics since 2010 in Canada (33).

Due to the growing controversy around the serological CHMP criteria and vaccine effectiveness, the licensing procedure of influenza vaccines has changed in Europe as of February 2017 and serological data alone (with abolition of the CHMP criteria) is no longer sufficient to conclude whether a vaccine is protective in a target population (34, 35). It is clear that for future next-generation influenza vaccines, both robust humoral and cellular immune responses are needed to offer a broader and longer-lasting protection against infection. Assessment of the cellular immunity after vaccination will thus become increasingly important in influenza vaccine trials as it has already been demonstrated that influenza-specific CD8^+^ T-cells can be an important correlate of protection against infection (36). First attempts to define cellular correlates of protection based on IFN-γ Enzyme-Linked ImmunoSpot (ELISpot) data have already been made but were unsuccessful (37–39).

## 3 Cell-mediated immunity after infection

A seroprevalence study of antibodies against influenza viruses (A/H3N2, A/H1N1 and B) in children of 0 to 7 years of age in the Netherlands showed that all children had acquired antibodies to at least one influenza virus by the age of 6 years while the highest attack rates were observed in children of 2 and 3 years (40). This shows that everybody is exposed to the influenza virus rather sooner than later in life. The first infection with an influenza virus induces an immune response that involves the mobilization and cooperation of numerous components of the immune system and leaves a long-lasting serological as well as cellular immunological imprint (41, 42).

**Figure 2:**
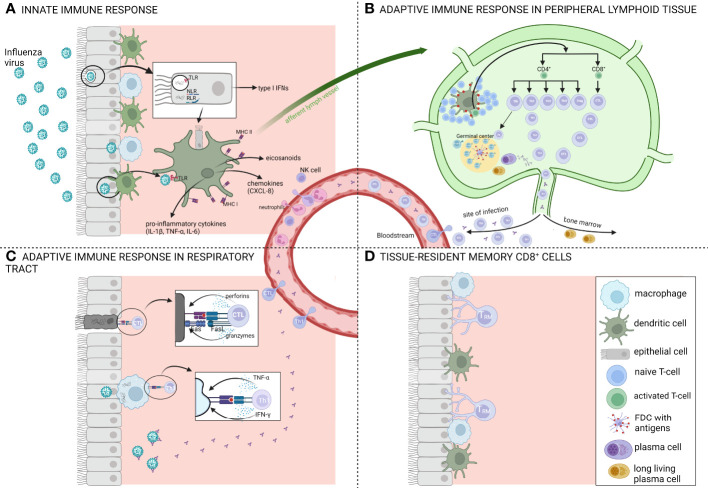
Schematic overview of the immune response after influenza infection. **(A)** Overview of the innate immune response which forms a first-line barrier in the mucosal surfaces. Viral RNA recognition by several pattern recognition receptors (PRR) like toll-like receptors (TLR), Nod-like receptors (NLR) and RIG-I-like receptors (RLR) leads to the secretion of type I interferons (IFNs) and pro-inflammatory cytokines such as IL-1β, TNF-α and IL-6. Antigen presenting cells (APCs) capture the antigens and migrate *via* the afferent lymph vessels to draining lymph nodes where they present the antigens to T-and B-lymphocytes. **(B)** Overview of the adaptive immune response in peripheral lymphoid tissue. T-and B-lymphocytes undergo a stepwise process of activation, proliferation and differentiation. Influenza infections typically induce a Th1-biased immune response. After activation, CD4^+^ and CD8^+^ T-cells migrate to the site of infection while long-lived plasma cells migrate to the bone marrow. **(C)** Overview of the adaptive immune response in the respiratory tract. Here, CD8^+^ T-cells or cytotoxic T-lymphocytes (CTLs) contribute to viral clearance by direct lysis of the infected cells *via* perforins and granzymes or *via* a Fas-dependent processes. **(D)** The respiratory tract once the infection is cleared, 90% to 95% of the influenza-specific T-cells undergo apoptosis and the remaining cells are destined to become long-lived tissue-resident memory T-cells (Trm). Figure created with BioRender.com.

After infection, both innate and adaptive immune responses are activated to combat and clear the virus (**Figure 2**). During the early phase of infection, viral replication and clearance are controlled by the innate immune system which forms a first-line barrier in the mucosal surfaces. Viral RNA within the infected cells is recognized by pattern recognition receptors (PRRs) which leads to the secretion of type I interferons (IFNs), pro-inflammatory cytokines, eicosanoids and several chemokines by macrophages, pneumocytes and dendritic cells. Type I interferons stimulate the expression of a variety of genes, known as IFN-stimulated genes, which induce an antiviral state. Pro-inflammatory cytokines and eicosanoids induce a local and systemic inflammation which cause fever and anorexia and the chemokines are involved in recruiting additional cells of the immune system such as NK-cells, neutrophils and monocytes. These NK-cells are responsible for the destruction of infected cells while the neutrophils and monocytes help in removal of these dead cells. Together with the resident alveolar macrophages, phagocytic clearance of virus-infected cells by recruited phagocytes is an important mechanism for viral clearance. However, if the virus persists and successfully establishes an infection, further support of the adaptive immune system is necessary to clear the virus (43).

The innate immune system is involved in shaping and regulating the induction of T-cell responses in the respiratory tract after infection. Antigen-presenting cells such as alveolar macrophages and dendritic cells, which reside just below the airway epithelial surface, capture the influenza antigens and subsequently migrate to draining lymph nodes to present antigen to naïve and memory T- and B-lymphocytes. These lymphocytes then undergo a stepwise process of activation, proliferation and differentiation (44, 45). After activation in the lymph nodes, CD4^+^ and CD8^+^ T-cells change the expression of homing receptors and migrate *via* several mechanisms to the site of infection. Here, CD8^+^ T-cells or cytotoxic T-lymphocytes (CTL) contribute to viral clearance by direct lysis of the infected cells *via* perforins and granzymes or expression of tumor necrosis factor (TNF) ligands (e.g. Fas ligand) of which the receptors (e.g. Fas) are expressed on infected cells. Both mechanisms result in apoptosis of the infected cells after which the cell debris is cleared by phagocytes such as macrophages and neutrophils (46). These CD8^+^ T-cells, which often target more conserved epitopes of internal proteins, offer a broader protection against different distinct influenza strains (47). The main viral antigens that induce a CD8^+^ response are the internal NP and PA proteins (48). CD4^+^ T-cells on the other hand mainly act indirectly by producing various cytokines which support B-cells and are crucial for CD8^+^ T-cells to acquire their killing capacity. However, a small subset of CD4^+^ T-cells has also direct cytolytic capacities *via* granzyme B and perforin dependent mechanisms and represents an important antiviral effector of cell-mediated immunity against influenza infection (27, 49). Different non-cytolytic subsets of CD4^+^ T-cells exist (Th1, Th2, Th17, follicular T-helper cells (Tfh) and regulatory T-cells (Treg)) which are all characterized by their unique cytokine patterns and functions. Influenza virus infection induces all CD4^+^ T-cell responses but there is a bias towards a Th1 response. The main function of Th1 cells is to enhance the pro-inflammatory cellular immunity while Th2 cells promote anti-inflammatory immune responses. Polyfunctional T-cells, T-cells with multiple functions such as degranulation of cytotoxic proteins and simultaneous production of multiple cytokines, are functionally superior over single-cytokine producing cells and are important in control of infection (50). In pregnant women, it was demonstrated that the proportion of polyfunctional CD4^+^ T-cells was inversely correlated with disease severity (51).

For the induction of a robust antibody response, the Tfh are critical. These cells are responsible for the formation and function of germinal centers in which the B-cells mature (45, 52). The neutralizing antibodies produced by these B-cells, which mainly target the (globular head of) HA and NA proteins of the virus, are critical for preventing serious symptoms and even death after infection. Despite the fact that yearly re-emerging viral strains can escape this humoral immunity by point mutations in the receptor binding region, pre-existing non-neutralizing antibodies can play a role in preventing serious disease and/or reduce mortality by activating antibody dependent cytotoxicity, cellular phagocytosis or complement system activation (53). Influenza-specific B-cell responses can also include less frequent responses against highly conserved epitopes of HA (stalk region), NA or M2e which are able to convey a broader cross-protection against different strains. Finally, B-cells produce cytokines which regulate the general immune response and can act as antigen presenting cells (APCs) to T-cells (46).

In general, natural infections induce a more diverse and broader protection compared to the vaccine-induced immune response. Recently, Miyauchi et al. demonstrated that after natural infection, IL-4 derived from follicular T-helper cells plays an essential role in the expansion of rare B-cells from the germinal center which is critical for generating broadly-protective antibodies (54). Natural infections are also able to induce cross-reactive NA antibodies while vaccination is less able to do so (55).

After clearance of the primary infection in the lungs, 90% to 95% of the influenza-specific T-cells undergo apoptosis and the remaining cells are destined to become long-lived memory T-cells. These cells are characterized by a high proliferative potential, a multipotent state, long-term survival and the capacity of self-renewal in the absence of the antigen. Upon re-infection, these cells undergo a rapid clonal expansion and differentiate to secondary effector T-cells in order to control the infection faster (45). Memory T-cells can be divided in three groups based on their location. A first group patrols the secondary lymphoid organs, the second circulates between the blood and non-lymphoid tissues while the third group resides in the lungs and the cells are called tissue-resident CD8^+^ T-cells (45). Recently, it has been demonstrated that lung-resident memory CD8^+^ T-cells are polyfunctional (antiviral/cytotoxic activity and chemokine/GM-CSF production) and have diverse T-cell receptor profiles indicating that they can immediately detect and combat different viral influenza strains *via* different mechanisms (56, 57). Lung resident T-cells however do not maintain long and wane over time due to an imbalance between apoptosis and lung recruitment of memory T-cells from the circulation (58). Measurement of these lung resident CD8^+^ T-cells in clinical trials is difficult as these can only be collected using invasive broncho-alveolar lavage or tissue biopsy. Different studies demonstrate the importance of memory T-cells in protection against re-infection of seasonal influenza (26, 27, 59, 60). A recent challenge study performed by Paterson *et al.* demonstrated that the number of influenza-specific memory CD8^+^ T-cells in circulation is inversely correlated with viral load (61). Both CD4^+^ and CD8^+^ T-cells induced during several influenza seasons also offer cross-protection against newly emerging pandemic strains as demonstrated during the 2009 pH1N1 pandemic (26, 62–64). Indeed, peripheral blood mononuclear cells (PBMCs) isolated from adults who have previously been infected are responsive to most influenza A virus proteins and primarily directed against the more conserved internal proteins such as NP and M1 (65, 66). This indicates that these PBMCs may convey broad cross-reactivity towards several influenza strains.

## 4 Cell-mediated immunity after vaccination

Since most people encounter the influenza virus and its antigenic components at very young age upon a first natural infection, the administration of an influenza vaccine at adult age and even during childhood rarely induces a primary immune response (40).

Classic inactivated split or subunit vaccines are administered intramuscularly and mainly generate a humoral immune response against the HA proteins of the strains incorporated in the vaccine. To a lesser extent, also NA-specific and non-neutralizing antibodies against more conserved epitopes such as the HA-stalk and NP region are generated (67). The magnitude of the vaccine-elicited IgG antibody response is determined by pre-existing immunity due to natural infection and/or previous vaccinations *via* follicular CD4^+^ T-cell responses, meaning that higher post-vaccination antibody responses are obtained in persons with pre-existing immunity at baseline (68). Follicular T-helper cells are an important mediator of the vaccine-induced humoral immunity as the increase of IgG antibodies is directly correlated with the expansion of Tfh in the circulation after vaccination (69). Also after intranasal administration of a LAIV, follicular T-helper cells are activated and proliferate in the nasopharynx-associated lymphoid tissue (NALT), a process that is essential for the induction of the anti-HA antibody response in the nasal mucosa (70). The activation and expansion of follicular CD4^+^ T-helper cells is thus crucial and directly associated with the antibody response after vaccination with either an intramuscular inactivated vaccine or an intranasal LAIV. In addition, LAIV vaccines also induce B-cell responses in the tonsils which correlate with the concentrations of systemic antibodies (38).

For several decades it was unclear whether inactivated influenza vaccines are able to generate robust antigen-specific T-cell responses. In 2006, a study demonstrated that after vaccination with a TIV, influenza-specific IFN-γ^+^ CD4^+^ and CD8^+^ T-cells were significantly increased in children aged 6 months to 4 years, but not in children of 5 to 9 years old nor in adults (71). This is probably due to the fact that older children and adults have already been exposed to the virus and/or vaccine(s) before and that post-vaccination fold changes of cellular immunity are inversely correlated with pre-vaccination levels or in other words, the higher the baseline level of pre-existing T-cells the lower the fold increase in IFN-γ^+^ T-cells after vaccination. Unlike TIV, LAIV vaccine was still able to induce significant increases in the 5 to 9 year old group, but not in adults (71). These results were confirmed by other studies that showed comparable low T-cell responses in TIV and LAIV vaccinated adults, but a significant induction of these responses in younger children receiving LAIV as demonstrated by IFN-γ-ELISpot and flow-cytometry based Intracellular Cytokine Staining assays (see further) (72, 73). The fact that LAIV is less effective in adults may be explained by the fact that older adults have higher pre-existing immunity acquired *via* previous natural infections and/or vaccinations which hampers infection by and replication of LAIV in the nasal cavity. Compared to inactivated vaccines, LAIV mimics more a natural infection and is able to generate broader T-cell responses and has thus the potential to generate a broader protection in younger children (38). In addition, LAIVs are also able to generate cross-reactive tonsillar CD8^+^ T-cell responses which recognize conserved epitopes from a broad range of seasonal and pandemic strains (74).

In general, vaccination offers a less strong immunization compared to natural infection. Indeed, fewer CD4^+^ memory T-cells or long-lived antibody-producing B-cells are generated after vaccination compared to natural infection and it is suggested that very high antigen levels are needed for vaccine-induced CD4^+^ effector T-cells to become memory T-cells (75). In 2020, a study concluded that infection elicits a stronger (polyfunctional) CD4^+^ response compared to vaccination in organ transplant recipients and thereby likely offers a better protection against reinfection (76). CD8^+^ T-cell development is determined by numerous factors such as the abundance, duration and tissue distribution of viral antigens and the form in which these antigens are presented to the APCs. All of these factors generating a strong CD8^+^ T-cell response are more favorable during natural infection (77). The composition of antigens to be ingested by APCs is also a key factor in the breadth of T-helper and B-cell responses. The antigen structure of native viruses is more complex compared to those present in vaccines which again leads to broadening of the immune response *via* epitope spreading (77). New strategies are thus necessary for the development of novel influenza vaccines which elicit stronger cross-reactive T-cell responses that mimic or even exceed the cellular immunity elicited by a natural infection.

## 5 Methods to assess cell-mediated immunity

A wide variety of techniques has been developed to analyze the magnitude and functional characteristics of the cellular immune response after infection or vaccination (**Table 2**). Traditional techniques are based on the detection of cytokine responses, phenotyping of T-cells, proliferation and cytotoxic activity of T-cells while more recent techniques are based on differential gene expression and activation of signal transduction pathways in activated immune cells. Here we discuss some assays which are used to study the immunogenicity of influenza vaccines or can be applied for the evaluation of new or improved vaccines. It is to be expected that one or more of these techniques will contribute to establish future cellular immune correlates of protection.

**Table 2:** Assays to evaluate cell-mediated immune responses.

Class	Assay	Cell type(s)	Cell Enumeration (Y/N)	Cell Phenotyping (Y/N)	Description	Read-out
Cytokine-based	ELISA	T-, NK- and NKT-cells	N	N	Detection of one cytokine secreted in supernatant by activated cells	Colorimetric
	Cytometric bead array/Luminex	T-, NK- and NKT-cells	N	N	Detection of multiple cytokines secreted in supernatant by activated cells	Flow cytometry
	IFN-γ ELISpot	T-, NK- and NKT-cells	Y	N	Detection of one cytokine (usually IFN-γ) which is secreted by activated cells	Colorimetric
	Fluorospot	T-, NK- and NKT-cells	Y	N	Detection of multiple cytokines secreted by activated cells	Fluorescence
Flow Cytometry-based	ICS	Depending on markers	Y	Y	Detection of multiple cytokines intracellular in activated cells	Flow Cytometry
	Cytokine secretion assay	T-, NK- and NKT-cells	Y	Y	Detection of one cytokine (usually IFN-γ) which is secreted by activated cells with simultaneous characterization of secreting cell (CD4, CD8, NK)	Flow Cytometry
	Tetramers	CD8^+^ T-cells	Y	Y	Detection of activated antigen-specific CD8^+^ T-cells *via* MHC I tetramers	Flow Cytometry
		CD4^+^ T-cells	Y	Y	Detection of activated antigen-specific CD4^+^ T-cells *via* MHC II tetramers	
	AIM	CD4^+^ T-cells	Y	Y	Detection of activated antigen-specific CD4^+^ T-cells *via* activation markers	Flow Cytometry
Proliferation	^3^H-thymidine	All dividing cells	N	N	Detection of proliferation by incorporation radioactivity	Scintillation counter
	BrdU	All dividing cells	Y	Y	Detection of proliferation by incorporation fluorescent signal	Colorimetric/Flow Cytometry
	EdU	All dividing cells	Y	Y	Detection of proliferation by incorporation fluorescent signal	Flow Cytometry
	CFSE	All dividing cells	Y	Y	Detection of proliferation by decreasing fluorescent signal	Flow Cytometry
	Alamar blue	All dividing cells	N	N	Reduction of substrate by mitochondria to a red colored product	Colorimetric
Cytotoxicity	^51^Cr release	Cytotoxic CD8^+^ T-cells and NK-cells	N	N	Detection of radioactivity released by target cells in supernatant	Scintillation counter
	LDH	Cytotoxic CD8^+^ T-cells and NK-cells	N	N	Detection of LDH released by target cells in supernatant	Colorimetric
	Calcein-AM	Cytotoxic CD8^+^ T-cells and NK-cells	N	N	Detection of calcein released by target cells in supernatant	Fluorometry
	CD107a	Cytotoxic CD8^+^ T-cells and NK-cells	Y	Y	Detection of CD107a	Flow cytometry
	Perforins/Granzyme B	Cytotoxic CD8^+^ T-cells and NK-cells	Y	Y	Detection of perforins and granzyme B	Flow cytometry
Transcriptomics	Gene expression scRNA-seq	PBMC	N	Y	Differential expression between activated and resting cells	Microarray RNA sequencing
Other	JAK-STAT pathway	PBMC	N	N	Increased activity of JAK-STAT pathway in activated cells	Microarray
	B-cell ELISpot	B-cells	Y	Y	Detection of antibodies secreted by activated cells	Colorimetric

### 5.1. Cytokine-based assays

#### 5.1.1. Detection and quantification of cytokines produced by activated immune cells *in vitro*

Cytokines are among the most commonly measured indicators of infection- or vaccine-induced immune responses. Enzyme-linked immunosorbent assay (ELISA) techniques, commonly used for the quantification of antibody concentrations and as such for the assessment of humoral immune responses, can also be applied for measurement of other proteins such as cytokines. Cytokine production by activated immune cells can be quantified in a variety of biological samples such as serum, plasma, bronchoalveolar lavage fluids and cell or tissue culture supernatants. ELISA methods are sensitive, precise and accurate. However, the technique is less often used for the assessment of cellular immunity in clinical studies as it is not able to determine the numbers and type of cells which are producing the cytokines (78). Standard ELISA kits measure only one cytokine at a time and require a relatively high sample volume. These shortcomings can be surmounted by using multiplex immunoassays which allow simultaneous quantification of multiple cytokines and/or chemokines. Examples of these are the BD^®^ cytometric bead array, Meso Scale Discovery^®^, AlphaPlex^®^ or Luminex^®^. These assays are based on different beads which are coated with specific capture antibodies on which the different analytes in the sample bind. Fluorescently conjugated detection antibodies are then added to the mixture after which flow cytometric analysis occurs (79).

#### 5.1.2. Detection and quantification of cytokine-producing immune cells activated *in vitro*

The ELISpot assay has the advantage over the aforementioned cytokine ELISAs that it determines the number of cells producing a given cytokine, with IFN-γ being the most frequently analyzed cytokine in influenza research. The technique was first developed to enumerate antibody-producing B-cells and was later adapted to quantify cellular immune responses using IFN-γ as marker protein. It is very sensitive and has been accepted as one of the most validated assays in human clinical research (80). An advantage is that antigen-specific cells can be expressed as a fraction of the total number of plated cells. PBMCs are incubated on a capture antibody coated plate and then stimulated with an antigen of interest (e.g. influenza NP, presented as a pool of overlapping peptides or other). In the presence of this stimulus, antigen-specific T-cells start producing cytokines (e.g. IFN-γ) which binds to the capture antibodies. In addition, bystander cells (T-cells, NK-cells, NKT-cells), which lack specificity for the antigen but are activated in a cytokine-dependent manner, may also start producing cytokines and are detected by the assay. After incubation (usually 16-20h), cells are washed away and the bound cytokine (cytokine of interest) is visualized *via* a conjugated secondary detection antibody and a colorimetric reaction (e.g. alkaline-phosphatase (AP)- 5-bromo-4-chloro-3’-indolyphosphate p-toluidine (BCIP) or horse-radish peroxidase (HRP)-tetramethylbenzidine (TMB) systems) (78, 79). The technique is widely used in both pre-clinical and clinical influenza research. ELISpot assays have been developed for the detection of cellular immune responses after influenza infection in both ferrets and mice (81, 82). The technique was able to demonstrate for the first time cellular immune responses after influenza vaccination in kidney transplant patients (83) and showed that atopic dermatitis patients elicit lower influenza-specific baseline T-cell responses (84). Alternatively, other cytokines or proteins can also be detected using ELISpot. For example, an assay targeted towards Granzyme B can be used for the quantification of cytotoxic T-cell responses. Salk et al. demonstrated that influenza-specific Granzyme B responses are increased after vaccination (85). For the detection of memory T-cells, a classic cytokine ELISpot can be used by extending the incubation period of the cells (10-14 days) (86).

A disadvantage of the classic ELISpot assay is that only one cytokine at a time can be analyzed. This can be overcome by the Fluorospot assay which uses multiple cytokine-specific capture antibodies and different secondary antibodies with various fluorophores. This technique allows for the detection of so-called double-cytokine positive cells and is increasingly being used in influenza research (61, 87–90). Another limitation is that the ELISpot assay does not reveal the phenotype of the cytokine-secreting cell while it has been demonstrated that cytokine production is not limited to T-lymphocytes but occurs *via* several cell types of the immune system (e.g. NK-cells) (91). These limitations can be overcome by using flow cytometric assays.

### 5.2. Flow cytometry-based assays

Flow cytometry allows for the characterization of the functional heterogeneity of T-cell responses as it provides information on both the phenotype and the cytokine production of the of the responding T-cells.

Intracellular Cytokine Staining (ICS) enables the detection and quantification of antigen-specific, low frequency, cytokine-secreting cells on a single-cell basis. As in ELISpot, the frequency of activated cells can be expressed as a fraction of the total population. PBMCs are stimulated with a specific antigen and a co-stimulus in the presence of a Golgi inhibitor (e.g. Brefeldin A or monensin) to prevent the secretion of translated cytokines so they remain intracellular in the cytoplasm. After staining of extracellular targets (e.g. CD3, CD4, CD8), the cells are fixed and permeabilized in order to permit intracellular staining of different cytokines with anti-cytokine antibodies which are conjugated to different fluorophores. Depending on the cytokine-specific antibody panels used, different T-cell subsets (Th1, Th2, Th17,…) can be identified (79). Due to its versatility, ICS is becoming the predominant method to assess cellular immune responses in influenza research. The complexity of the procedure, high reagent and equipment costs and the expertise needed for correct data acquisition and interpretation, are just a few disadvantages that limit a widespread use of this technique (78). Another approach is the cytokine secretion assay in which the secreted cytokine is retained on the surface of the secreting cell with the help of bispecific antibodies. One antigen binding site interacts with a cell surface molecule (e.g. CD45) while the other binds to the cytokine secreted by that cell. This cytokine is then revealed by a specific detection antibody and flow cytometric analysis (92). This assay has the advantage that it measures the actual secreted cytokines while ICS may also measure cytokines which would not necessarily be secreted and thus are of lesser biological importance.

The tetramer assay is a very specific and sensitive method to detect antigen-specific CD4^+^ and CD8^+^ T-cells. It shows less intra-assay variation, better precision and linearity compared to the ICS and ELISpot assays. It is based on tetramers of synthetic biotinylated MHC Class I (for CD8) and Class II (for CD4) molecules which are conjugated to fluorescently labeled streptavidin molecules. These complexes are loaded with antigen-specific peptides which then bind to the CD4^+^ or CD8^+^ T-cells of interest *via* the T-cell receptor. This approach increases the avidity for epitope specific interactions and has already extensively been used in influenza research (79, 93, 94). Detection of CD4^+^ T-cells is more complicated seen the higher diversity of MHC II allelic variants, but it is not impossible. Ye et al. were able to detect influenza-specific CD4^+^ T-cells in healthy volunteers (95). This approach has also been used to demonstrate the expansion of specific CD4^+^ T-cell responses after influenza vaccination (96, 97). In general, the tetramer assay is more suitable for pre-clinical research due to the extensive genetic polymorphisms of the HLA molecules in humans (78).

Alternatively, activation induced surface markers (AIM) can be used for the detection of activated antigen specific immune cells with minimal bystander activation effects. A variety of these assays have been developed for the detection of antigen specific CD4^+^ T-cells. Examples of CD4 activation markers are CD40L, CD69, OX40, CD25, PD-L1 and 4-1BB (98).

### 5.3. Proliferation-based assays

As discussed earlier, during an adaptive immune response activated B and T-cells start to proliferate, a reaction that can be detected and quantified *via* different assays. Most *in vitro* assays make use of the incorporation of a marker molecule in the newly-formed DNA of dividing cells. These marker molecules can be either radio-active compounds or fluorophores that are measured by a scintillation counter or flow cytometer, respectively. The very first proliferation assay was the ^3^H-thymidine assay in which this radio-active nucleoside is incorporated in the DNA of dividing cells which results in an increasing radio-active signal measured with a scintillation counter. Results are typically reported as a stimulation index in which the counts measured in stimulated cells are divided by those measured in unstimulated control culture. This technique was extensively used in the 80’s and 90’s but was progressively replaced by non-radioactive alternatives such as the 5-bromo-2’-deoxyuridine (BrdU) assay. This assay is based on the antibody-mediated detection of BrdU which is incorporated in the DNA of dividing cells. These antibodies can be either conjugated to a fluorophore and measured by flow cytometry or conjugated to an enzyme (i.e. AP or HRP) and measured by a colorimetric reaction (99). A disadvantage of this alternative is that incorporation of this chemically modified thymidine analog can induce errors in the dividing cells and hence influence biological functions such as proliferation (100). An earlier alternative is the Alamar blue assay in which the blue dye is reduced to a red colored product by mitochondrial active cells which is measured by an ELISA reader (101). A more recent method is based on the incorporation of EdU (5-ethanyl-2’-deoxyuridine) into DNA and the visualization of a fluorescent azide after a copper(I) mediated reaction *via* fluorescence microscopy or flow cytometry. The main advantage of this technique is that it is compatible with other fluorescent markers and thus allows for identification of the dividing cells while all other assays are not able to distinguish between the different dividing cells (102). A last assay described here is the CFSE assay. In this method, long-lived intracellular proteins are covalently labelled with carboxyfluorescein succinimidyl ester (CFSE) before the cells are stimulated. After each cell division, the fluorescent signal is equally divided over both daughter cells that will generate a signal with half the intensity of the mother cell. Each cell division can be assessed by measuring the corresponding decrease in cell fluorescence *via* flow cytometry. Like the EdU assay, the CFSE method is also compatible with other fluorochromes and enables identification of the dividing cells (103). In addition, this method can also be applied to study *in vivo* activation and proliferation of T-cells that have been CFSE-labeled *ex vivo* and re-introduced into a living (non-human) host (104). All these fluorescent-based assays have a lower sensitivity compared to the ^3^H-thymidine assay which is however, for evident reasons, no longer the preferred method (105).

### 5.4. Cytotoxicity-based assays

Functional cytotoxic assays quantify the ability of CD8^+^ cytotoxic T-cells and NK-cells to lyse virus infected cells, a function of the immune system which is important for viral clearance. A wide variety of cytotoxic assays exists and can be classified in different categories such as dye exclusion methods (e.g. Trypan blue), fluorescent DNA binding dye assays (e.g. propidium iodide), metabolic activity assays (e.g. MTT assay) and markers that leak out of dying/dead cells in the supernatant (e.g. ^51^Cr, lactate dehydrogenase (LDH)) (106, 107). In both the ^51^Cr and LDH release assays, cytotoxicity is measured in cultures wherein, depending on the cell types examined, different ratios of effector to target cells are used. The difference between both assays is that LDH is naturally present in the cells while ^51^Cr has to be loaded into the target cells before these are confronted with the effector cells. Non-radioactive alternatives for ^51^Cr are the Calcein-AM or fluorolysometric-CTL assays which measure the release of fluorescent compounds in the supernatant (108). While ^51^Cr is bound to proteins in the cytoplasm, Calcein-AM is taken up by live cells where the AM group is detached *via* cytoplasmic esterase activity to generate the fluorescent calcein that is retained in the live cell and released after necrosis/apoptosis of this cell (107). In addition, a number of flow cytometric approaches can be used for the detection of apoptosis in the target cells (Annexin V staining or caspase activation) (109). In general, these assays measure the elimination of target cells but are unable to directly measure the number of cytotoxic effector cells (78).

This is overcome by flow cytometric analysis using CD107a staining which allows for the detection of activated cytotoxic CD8^+^ T-cells and NK cells. CD107a is a lysosomal-associated membrane protein (LAMP-1) which is present on the lipid bilayer of lytic granules. Therefore, membrane expression of this protein is a marker for cytotoxic degranulation of cells and it has been demonstrated that this expression (as measured by flow cytometry) is directly correlated to increased cytotoxicity (as measured by a fluorolysometric assay) (110, 111). This method is increasingly being used in influenza infection and vaccine research (112–115). Other methods are the detection of perforins and granzyme B by flow cytometry (but also by ELISA or ELISpot) which are also indirect markers for cytotoxicity (85, 116, 117).

### 5.5. Transcriptomics

A more recent approach to study pathogen- or vaccine-induced responses is the measurement of differential gene expression patterns between activated and non-activated immune cells which can be investigated by microarray or more recent next generation sequencing technologies (118). Measuring the effects of vaccination on gene expression can be used to assess both the safety and immunogenicity of investigational vaccines (119, 120). Several studies demonstrate that gene expression is altered after natural influenza infection or vaccination. The inter-individual variability observed after natural infection or vaccination can be explained by inter-individual differences in gene expression in airway epithelial cells and immune cells (121). Alcorn et al. showed that these gene expression changes are transient in time with different differentially expressed genes at three and seven days after vaccination compared to pre-vaccination expression profiles (122). Another study demonstrated that these expression changes are sex-specific which can explain the sex-dependent differences in immune responses after vaccination. Based on gene expression data, it is suggested that women generate a stronger early immune response within 24 hours after vaccination while men are able to maintain a longer response (123). In addition, the difference in immune responses between LAIV and inactivated vaccines can also be explained at the level of gene expression (124). de Armas et al. even suggested to use transcriptional data as a correlate of vaccine response in HIV-infected children (125). A recent study demonstrates that influenza vaccines are also able to epigenetically remodel cells of the innate immune system (monocytes and dendritic cells) which is associated with a higher expression of antiviral genes (126).

One of the disadvantages of this bulk sequencing is that the behavior of all cells is averaged within the population and that antigen-specific responses are ‘diluted’ by non-affected cells. A limitation which is not present in single-cell approaches. Single-cell RNA sequencing (scRNA-Seq) is able to characterize gene expression on single-cell level and shows great promise in vaccinology by better understanding host-pathogen interactions (127). One of the first studies using scRNA-Seq in influenza-vaccine research was performed in 2019 where Neu et al. demonstrated that vaccine-negative plasmablasts were transcriptionally distinct from antigen-induced plasmablasts (128).

Another approach to identify cellular immune responses after vaccination is the analysis of signal transduction pathways. A recently developed mRNA-based method uses JAK-STAT signal pathway activity to quantitatively measure cellular immune responses using microarray data. In this assay, a higher pathway activity is associated with a stronger adaptive immune response. This assay can be used for the analysis of whole blood samples or isolated PBMCs. Activity of the JAK-STAT1/2 pathway is elevated in whole blood samples of influenza infected patients. However, after vaccination with a TIV, no increases in activity are observed which can be explained by the fact that TIV do not elicit a strong cellular immune response (129). Alternatively, phosphorylation-based signaling pathways can also be analyzed using phospho flow-cytometry in which phospho-specific antibodies target epitope sites that are phosphorylated upon activation. Combined with phenotypic antibodies, this technique allows for the analysis of intracellular phosphorylation events on single-cell level in a mixed cell population (130, 131).

Besides transcriptomics, other biological systems such as metabolomics, proteomics, lipidomics and glycomics can be employed to investigate influenza vaccine responses (132).

### 5.6. B-cell assays

B-cell activity can be indirectly detected *via* measurement of antibody titers, but also by using ELISpot which uses plates that are coated with the antigen of interest (e.g. HA, NA, NP) on which the antibodies produced by the B-cells bind. This approach is often used for detection of memory B-cells and/or plasmablasts (133). Using this technique, Zhan et al. demonstrated that patients with common variable immune deficiency (CVID) showed impaired (memory) B-cell responses after vaccination which matches with the impaired antibody titers observed (134). In addition, flow-cytometry can also be used for the detection of circulating influenza-specific B-cells using recombinant HA as stimulating antigen. The advantage of this technique is that it allows for the identification of B-cell subsets such as follicular, germinal center, plasmablasts and memory B-cells by using multiple markers (135).

### 5.7. Standardization of assays

It is clear that using these methods may contribute to the establishment of cellular immune correlates of protection in influenza vaccine trials. However, the diversity of these assays, the large degree of variation in their execution and the way data are analyzed and reported, remain a considerable challenge for comparing cellular immunogenicity data of influenza vaccines between different trials. The lack of standardized protocols and identification of positive cut-off values leads to a high interlaboratory variability. For example in the IFN-γ ELISpot assay, parameters that are variable among laboratories are: incubation times, seeding density, concentration of antigen, antigen type (whole protein, virion or peptide pools), use of fresh or cryopreserved PBMCs and differences in equipment and reagents. As a result, even baseline ELISpot responses vary greatly between different studies. Therefore, it can be more informative to use fold changes (pre-vaccination vs post-vaccination) rather than absolute ELISpot data to define correlates of protection using the IFN-γ ELISpot assay. These data then need to be generated in the context of human challenge studies or studies where natural influenza infections are monitored (79). Similar discrepancies exist when using the ICS assay with also a great technical variability between laboratories. The limited number of human influenza challenge studies and the non-existence of large-scale prospective population studies further explain why no cellular correlates of protection have yet been defined.

Initiatives such as FLUCOP aim at developing standardized methods to assess cellular immunity. These may eventually reduce the interlaboratory variation and hereby facilitate the discovery of cellular correlates of protection and the development of cross-protective universal influenza vaccines. One of the main goals of the project was the development of standardized protocols for PBMC preparation and cryopreservation, IFN-γ ELISpot, ICS and data analysis and reporting (136). Similar initiatives exist for the standardization of serological assays such as the HI, SRH and microneutralization assays (i.e. FLUCOP and CONSISE) (137).

## 6 Cell-mediated immunity in (next-generation) influenza vaccine trials

As of February 2017, the European Medicines Agency’s (EMA) committee for human medicines (CHMP) implemented new guidelines for market authorization of novel influenza vaccines (34). Measurement of cell-mediated immunity (CMI) is strongly encouraged, especially in older adults whom as mentioned above, conventional correlates of protection do not really apply (138). CMI assessment should therefore be performed for every new influenza vaccine in clinical development as this is essential to obtain a clear understanding of the immune response in the population of interest (35).

Several novel or improved influenza vaccines are currently under investigation, such as vaccines formulated with potent adjuvants, vaccines targeting more conserved viral proteins, viral-vectored vaccines and mRNA based vaccines (139). Better influenza vaccines are much needed to improve the overall protective efficacy, in particular in older adults by overcoming immunosenescence and to offer a broader and more durable protection in all age groups.

This chapter provides an overview of a selection of clinical trials in which the cellular immunogenicity of novel or improved influenza vaccines has been examined.

### 6.1. Cell-mediated immunity induced by adjuvanted influenza vaccines

Adjuvants are vaccine components that enhance the magnitude, breadth and durability of the immune response (140). Adjuvantation of influenza vaccines has been applied to increase the immunogenicity of trivalent (and later quadrivalent) influenza vaccines in older adults to restore their weaker immune responses as a consequence of immunosenescence. The first and most widely used adjuvants are aluminium salts, but it has been demonstrated that these adjuvants are not effective when combined with influenza antigens (141). The first novel type of adjuvant authorized for widespread use in influenza vaccines were oil-in-water (o/w) emulsions (142). These achieve a superior immune response post-vaccination by releasing specific cytokines like IL-5 and IL-8 at the site of injection which increase antigen uptake by APCs and induce a mixed Th1/Th2-oriented immune response (143). Three o/w emulsions that have already been investigated for use in commercial influenza vaccines are AS03 (GlaxoSmithKline; GSK), MF59 (Novartis), and AF03 (Sanofi Pasteur). AS03 was been marketed for use in a pandemic H1N1 vaccine in 2009 (Pandemrix^®^). MF59 is approved for use in seasonal flu vaccines (Fluad^®^) while AF03 was only evaluated in clinical studies of influenza vaccine candidates (144). Interestingly, several studies demonstrate the generation of higher antibody titers and a larger expansion of vaccine-specific CD4^+^ T-cells after vaccination with an MF59 adjuvanted TIV compared to a non-adjuvanted TIV, in young children, adults and older adults (145–148). A recent study demonstrated that a high antigen dose and MF59 adjuvantation also resulted in increased polyfunctional CD4^+^ and CD8^+^ T-cell responses in older adults compared to a standard dose of the seasonal inactivated vaccine (149). Other studies in both children and adults showed that CD4^+^ T-cell and B-cell responses were stronger in the AS03-adjuvanted group compared to the non-adjuvanted group (150–153). These studies demonstrate the superiority of adjuvanted over non-adjuvanted vaccines in both humoral and cellular responses, thus allowing for antigen sparing and a broader cross-reactive protection (145–148, 154). During the flu season 2008-2009, a large randomized phase 3 trial was done in adults aged 65 and older to compare the protective efficacy of AS03-adjuvanted versus non-adjuvanted TIV. The study showed that an AS03-adjuvanted TIV is not superior to a non-adjuvanted TIV for the prevention of all types of influenza in older adults. However, the data suggested that the benefit of influenza vaccination in elderly people might vary depending on influenza subtypes. In a *post-hoc* analysis, the highest increase in protection (12%) of the adjuvanted TIV was observed against influenza A H3N2. CMI was not explored in this study (155). Other more recent studies have meanwhile demonstrated that adjuvanted QIV could offer a reduction in costs for society by reducing hospital admissions and deaths compared to a high dose QIV (156, 157).

Since the marketing of these first o/w adjuvants, many more substances are being considered as potential adjuvants for influenza vaccines that could induce strong humoral but also cell-mediated immunity. However, none of these have been licensed yet and most of them are still in the preclinical stage (158).

Another promising adjuvant is Matrix-M, a saponin-based adjuvant patented by Novavax (159, 160). Matrix-M has a potent and well-tolerated effect by stimulating the entry of APCs into the injection site and enhancing antigen presentation in local lymph nodes. It has recently been licensed in a recombinant nanoparticle vaccine against SARS-CoV-2 (Nuvaxovid™). In a pivotal phase III trial (NCT04120194) in older adults (≥65 years), a recombinant quadrivalent nanoparticle influenza vaccine with Matrix-M, called NanoFlu™, has been shown to induce non-inferior immunogenicity in terms of HAI response and a significant greater induction of cellular immunity in comparison with a classic quadrivalent vaccine (Fluzone quadrivalent) seven days after vaccination as measured by ICS (IL-2, CD40L, IFN-γ and TNFα). The fraction of both single positive as well as poly-positive CD4^+^ T-cells was higher in the adjuvanted vaccine group (160). FDA has granted Fast Track designation for NanoFlu™ in older adults.

### 6.2. Cell-mediated immunity induced by vaccines targeting conserved influenza proteins

As discussed above, the effectiveness of currently licensed influenza vaccines largely depends on a good match between the vaccine strains and the circulating strains. When a mismatch occurs, the vaccine’s ability to protect against antigenically different circulating strains is reduced. To circumvent this major shortcoming, novel vaccines are being developed that target more conserved influenza proteins, or conserved epitopes thereof, rather than the highly variable globular head of HA. This approach aims to induce a broader immune response, not only antibody-mediated but also cellular, with a higher potential to convey cross-protection against drift variants and ideally also shifted strains with pandemic potential (161–163). One of those well-conserved proteins that displays very limited antigenic variation is the influenza nucleoprotein (NP) (164). Recently, several human observational studies and pre-clinical studies reported that CD4^+^ and/or CD8^+^ T-cells specific to NP epitopes could provide additional protection (26, 27, 165, 166). OVX-836, a recombinant influenza vaccine developed by Osivax (Lyon, France), contains seven copies of the target antigen (NP) fused to the patented heptameric oligomerization domain (oligoDOM^®^) (167). Oligomerization of antigens has been proven to induce improved humoral and cellular immune responses in animals (168, 169). In a phase I dose-finding study, several formulations of OVX-836 were investigated for cellular immunity. All dose levels were able to induce cellular immune responses on day 7 post-vaccination, as demonstrated by IFN-γ ELISpot (167). A phase II study evaluating the immunogenicity, safety and reactogenicity of different dose levels of OVX-836 compared to the QIV Influvac Tetra™ confirmed these ELISpot results and showed an increase of CD4^+^ polyfunctional T-cells in a dose-dependent manner (170).

A second novel influenza vaccine candidate is the Flu-V recombinant vaccine, developed by PepTcell (SEEK, London, UK). It contains four synthetic polypeptides that cover regions from the conserved NP, matrix proteins M1 and M2 of both human and animal influenza A and B strains and is administered with a potent Montanide ISA-51 water-in-oil emulsion adjuvant (171). In the Phase I trial, PBMCs from participants that received the study vaccine demonstrated a 2-fold increase in IFN-γ production three weeks after vaccination as measured by ELISA (172). This was confirmed in the subsequent Phase II study. Administration of a single dose of adjuvanted Flu-V resulted in a strong Th1-skewed cellular immune response, as measured by both ELISA and ICS (173).

A third vaccine candidate directed against conserved influenza epitopes was the Multimeric-001 (M-001) vaccine developed by BiondVax Pharmaceutical Ltd. (Israel). M-001 is a synthetic recombinant protein specifically designed to activate both cellular and humoral immunity. It contains 3 repetitions of 9 B- and T-cell epitopes from the HA, M1, and NP viral proteins from both influenza A and B strains. In the initial phase I trial, the M-001 vaccine was shown to induce cellular responses as demonstrated by the ^3^H-thymidine lymphoproliferation assay and secretion of IL-2 and IFN-γ by ELISA upon stimulation of PBMC with M-001, different influenza virus strains or T-helper epitope peptides derived from the NP and HA proteins (174). The phase II trial results demonstrated that M-001 was able to trigger a significant increase in polyfunctional CD4^+^ T-cells (ICS) when it was used as a primer for subsequent QIV vaccination (unpublished results) (175). However, a pivotal phase III study later that year demonstrated no significant difference between vaccine and placebo groups in the reduction of flu illness and severity and therefore failed to meet both the primary and secondary efficacy endpoints (unpublished results) (176).

### 6.3. Cell-mediated immunity induced by viral vector-based influenza vaccines

Since the 1990s, viral vector-based vaccines have been investigated in a wide range of diseases including bacterial and viral infections, and even some cancers (177, 178). Initially, these vaccines showed great promise in murine models but had difficulty reproducing these results in larger animal studies (179, 180). A Modified Vaccinia Ankara (MVA) vectored vaccine (MVA-NP+M1) encoding for the full-length NP and M1 antigens from a H3N2-strain virus has been developed by Vaccitech, a University of Oxford spin-off. MVA has been known to generate strong T-cell responses to several antigens, including malaria, tuberculosis, and HIV. In a phase I trial, Berthoud et al. showed that the MVA-NP+M1 vaccine induced T-cell responses within 1 to 3 weeks after administration as measured by IFN-γ ELISpot and ICS (181). In adults aged 50 to 59 years, increased T-cell responses remained significant until one year after vaccination. In subjects aged 70 years and older, induced T-cell responses only remained significant until 3 weeks post-vaccination, which could indicate a lower efficacy in this age group. Both CD4^+^ and CD8^+^ T-cells were increased with also an expansion of pre-existing CD8^+^ T-cells (182).

A phase IIb study was conducted where participants received the MVA-NP+M1 vaccine after seasonal QIV vaccination. Comparable levels of CD4^+^ and CD8^+^ T-cells were seen as in the earlier phase I trials, but the vaccine regimen was unable to decrease total viral shedding, symptom score, or the incidence of influenza-like illness after infection (183). The authors hypothesized that these unexpected results might be caused by the fact that intramuscular vaccination only increases peripheral T-cells without increasing the resident-memory T-cells residing in both the upper and lower respiratory tract which are important for the local mucosal immune response (183, 184). Next, a heterologous vaccination regimen of the MVA-NP+M1 in combination with a chimpanzee adenovirus ChAdOx1 NP and M1 (ChAdOx1 NP+M1) was investigated in a following phase I study. This adapted scheme also showed promising results with significant increases in T-cell responses after vaccination and showed slightly higher IFN-γ ELISpot responses compared to the single MVA-NP+M1 dose regimen (185).

Besides the MVA vector, adenoviruses are another potential vector for influenza vaccines (186). As these viruses belong to the same family as some naturally occurring respiratory viruses, they can, when administered intranasally, mimic natural infection (e.g. penetrate nasal mucosa) in a very similar way as influenza viruses (187). These vectors are known to have an excellent safety profile and induce a balanced humoral and cell-mediated immune response when used as a vaccine platform (186). Altimmune Inc. has recently developed a novel influenza vaccine for intranasal administration named NasoVAX (188). By incorporating a HA gene into a replication-deficient adenovirus, this adenoviral vector can transduce the HA gene into cells of the nasal mucosa, leading to local expression of the encoded HA protein and presentation to resident CD8^+^ T-cells through the HLA class I antigen processing machinery (187, 188). The proof of concept was confirmed in a phase I trial where (humoral) immune responses were weak but measurable (189). In a subsequent phase II trial, the intranasal NasoVAX vaccine was able to induce a dose-dependent and significant T-cell response as measured by IFN-γ ELISpot eight days after vaccination. However, it remains unclear whether NasoVAX can induce resident memory T-cells in the respiratory tract as this was not within the scope of the trial (188).

## 7 Conclusion

It is clear that current licensed influenza vaccines do not always offer the protection needed to prevent illness after influenza infection. Antigenic drift of the virus necessitates repetitive yearly vaccinations to stay protected and even then, vaccine efficacy is far from satisfactory and largely dependent on the match between the vaccine strains and circulating strains.

Adjuvanted or next generation universal influenza vaccines that target more conserved epitopes and elicit broader cross-reactive T-cell responses could overcome these shortcomings of the current vaccines. In future trials, assessment of induced cellular immune responses will thus become increasingly important. However, due to the large variability in both the technical aspects of the assays and representation of the data, it remains challenging to identify cellular correlates of protection for influenza vaccines.

The FLUCOP project was initiated to support the development of new influenza vaccines by tackling the issue of assay diversity *via* the harmonization of the most widely used assays to measure humoral and cellular immune responses elicited by influenza vaccines and developing and evaluating the usefulness of new analytical methods.

## Author contributions

YJ and JJ wrote the manuscript. GW, FC, GL-R, and IL-R revised the manuscript. JJ generated the figures. All authors approved the manuscript.

## Funding

The FLUCOP project is supported by the Innovative Medicines Initiative Joint Undertaking under grant agreement 115672, resources of which are composed of financial contribution from the European Union’s Seventh Framework Programme (FP/2007-2013) and EFPIA companies’ in kind contribution.

## Conflict of interest

The authors declare that the research was conducted in the absence of any commercial or financial relationships that could be construed as a potential conflict of interest.

## Publisher’s note

All claims expressed in this article are solely those of the authors and do not necessarily represent those of their affiliated organizations, or those of the publisher, the editors and the reviewers. Any product that may be evaluated in this article, or claim that may be made by its manufacturer, is not guaranteed or endorsed by the publisher.
